# Preventing acute diverticulitis. any roles for non-absorbable antibiotics? in search of evidence: a systematic review, meta-analysis, and trial sequential analysis

**DOI:** 10.3389/fgstr.2023.1170271

**Published:** 2023-05-01

**Authors:** Maurizio Koch, Alberto Enrico Maraolo, Giuseppe Natoli, Salvatore Corrao

**Affiliations:** ^1^ GI & LIver Unit, General Hospital S.Filippo Neri, Club for Evidence Based Gastroenterology & Hepatology, Rome, Italy; ^2^ First Division of Infectious Diseases, Cotugno Hospital, AORN Ospedali dei Colli, Naples, Italy; ^3^ Department of Clinical Medicine and Internal Medicine Unit, National Relevance and High Specialization Hospital Trust ARNAS Civico, Di Cristina, Benfratelli, Palermo, Italy; ^4^ Department of Health Promotion Sciences, Maternal and Infant Care, Internal Medicine, and Medical Specialties [PROMISE], University of Palermo, Palermo, Italy

**Keywords:** diverticular disease, meta-analysis, non-absorbable antibiotics, diverticulitis, trial sequantia

## Abstract

**Background:**

Hospital admissions for diverticulitis, a complication of diverticular disease, are very much on the increase. Prevention of diverticulitis could cut costs and save lives.

**Aims:**

To identify whether the risk of the first episode of diverticulitis (primary prevention) or recurrence of diverticulitis (secondary prevention) can be reduced in patients with diverticular disease using non-absorbable antibiotics (mainly rifaximin).

**Methods:**

The studies were identified by searching PubMed and CENTRAL from 1990 to May 2022. The methodological quality of each study was also evaluated. The outcome of the meta-analysis was the occurrence of a first or subsequent episode of diverticulitis. In addition, a trial sequential analysis was performed to evaluate whether the results would be subject to type I or type II errors.

**Results:**

Primary prevention: the risk difference was statistically significant in favor of rifaximin (-0,019, or -1.9%, CI -0,6 to -3,3%). There was no evidence of heterogeneity *(I^2^* 0%). At one year, two years, and eight years of age, the NNT was 62, 52, and 42, respectively. The level of evidence had a moderate degree of certainty. Secondary prevention: the risk difference was statistically significant in favor of rifaximin (- 0,24, or -24%, CI -47 to -2%). There was evidence of heterogeneity (*I^2^* 92%); NNT resulted in 5. The grade level was low.

**Conclusions:**

Rifaximin can lower the risk of a first episode of diverticulitis. However, the cost-benefit ratio currently appears too high. Rifaximin could also reduce the risk of a second episode, but the quality of the evidence is low.

**Systematic review registration:**

https://www.crd.york.ac.uk/prospero/, identifier CRD42022379258.

## Introduction

Doctors and patients face several decisions in appropriate management after the first episode of left colon diverticulitis. Episodes of acute diverticulitis are generally uncomplicated (causing only localized inflammation), but complicated diverticulitis, defined as inflammation associated with an abscess, fistula, hemorrhage, or perforation (1–3), occurs in about 12% of cases (4). Relapses occur in about 8% to 36% of patients between 1 and 10 years (2, 3), and prevention is of great importance. Evidence for the use of various pharmacological and surgical interventions to prevent diverticulitis recurrence has evolved over time (5–11).

The purpose of this meta-analysis is to identify whether the risk of the first episode of diverticulitis or of recurrence of diverticulitis in symptomatic uncomplicated diverticular disease (SUDD) can be reduced using non-absorbable antibiotics (mainly rifaximin), through the identification of published randomized and observational studies.

We followed GRADE guidance 24 and used a framework that considers the certainty of evidence from randomized and non-randomized studies then in an integrative fashion (12).

A first analysis of the evidence gathered for the role of non-absorbable antibiotics based only on symptoms in SUDD appeared in 2011 (13). One of the authors is in the editorial team of this paper.

According to the 2022 latest American guidelines for internists, evidence is very uncertain (insufficient) for treatments to prevent recurrence (like probiotics, combinations of mesalamine and rifaximin, combinations of mesalamine and probiotics, and burdock tea) (14).

The most studied drug for preventing diverticulitis is mesalamine. However, the latest American guidelines definitively exclude a role for this agent in preventing relapses (*strong recommendation; high-certainty evidence)* (14), following the results of the last meta-analysis (15).

This meta-analysis could help figure out if nonabsorbable antibiotics might play a role in lowering the risk of diverticulitis and, if so, what studies should be done.

Details of our systematic review are registered in the PROSPERO database under the number [379258].

### Materials and methods

The general recommendations of the PRISMA review were considered for the meta-analysis (16, 17).

### Literature search

The studies were identified by searching PubMed, and the Cochrane Central Register of Controlled Trials from 1990 to May 2022.

#### The search strategy for both databases used the following string

((((((diverticulitis OR diverticular)))) AND (((recurrence OR relapse OR rehospitalization))))) NOT ((((((diverticulitis OR diverticular)))) AND (((recurrence OR relapse OR rehospitalization)))) AND ((((clinical[Title/Abstract] AND trial[Title/Abstract]) OR clinical trials as topic[MeSH Terms] OR clinical trial[Publication Type] OR random*[Title/Abstract] OR random allocation[MeSH Terms] OR therapeutic use[MeSH Subheading])))).

#### The search strategy was completed on May 30, 2022

The identified records were screened by titles, abstracts, and keywords. Papers with potential eligibility were then obtained for full-text review. No language limits were imposed. We supplemented the electronic search by scanning the reference lists of relevant publications, including review articles and guidelines. When published data were insufficient for our analyses, additional details were sought from the investigators of the corresponding clinical trials.

The flow chart of the items identified and those then eliminated was developed with the help of Prisma 20202 software (https://doi.org/10.1002/cl2.1230).

### Study selection

The PICO question format (18) was used to figure out who was eligible first.

- Patients: patients with symptomatic, uncomplicated diverticular disease (SUDD) who have never had diverticulitis or have only had it.
- Intervention: long term administration of rifaximin. The allowable dose was 800 mg per day in cycles of 7–10 consecutive days per month.
- Comparators/controls: standard of care, placebo, or mesalamine.
- Outcome: the occurrence of a diverticulitis episode, whether first-time or recurring.
- Study design: randomized, non-randomized, and observational studies if peer-reviewed and published in full.

### Definitions

All patients suffered from SUDD, defined as a syndrome characterized by recurrent abdominal symptoms attributed to diverticula in the absence of other macroscopically evident alterations other than the presence of diverticula.

Diverticulitis was predefined as abdominal pain attributed to diverticular disease *and* one of the following findings: (1) requiring hospitalization or surgery; or (2) described as acute and presenting with fever, and/or being evaluated with computed tomography. Prevention of the first episode of diverticulitis was considered “primary prevention” (PP) when the complication had never appeared previously or “secondary prevention” (SP) when it appeared after the first episode. All articles passed through a systematic review by a team of 3 physicians (MK, SC, and AEM), and methodological criteria and the results were recorded. Studies that fulfilled the inclusion criteria were evaluated by a blinded review done independently by the same 3 authors to tabulate subject demographics, study design, definition of outcomes, and frequencies of diverticulitis using a standardized data form. Disagreement was resolved by consensus.

### Quality appraisal

The Newcastle-Ottawa Scale (NOS), a tool for rating the quality of non-randomized research, was used by the same authors to assess the methodological quality of each study (19). NOS has three areas: selection, comparability, and outcome. A maximum of 13 stars can be assigned.

The Robvis web app was used to measure the risk of bias (Rob 2) (20, 21) in randomized controlled trials.

The Grading of Recommendations Assessment, Development, and Evaluation (GRADE) tool was used for a global evaluation of the body of evidence in the systematic review (22). The level of confidence is determined by the study design (high for RCTs, low for observational studies), whereas reasons for lowering confidence are based bias risk, imprecision, or inconsistency (23). Discrepancies in ratings were resolved between the authors.

### Statistical analysis

The DerSimonian and Laird method was used to compare and summarize the outcomes of each study (24). We used the random effect model since it is more conservative. The risk difference (RD), i.e., the difference in event rates between the treatment and control groups, was used to measure the prevention effect. Confidence intervals (CI) were calculated at 95%.

Along with the pooled effect sizes, a prediction interval (PI) was given. This showed how the effects of the treatment changed in different settings, as well as what effects to expect in future patients (25).

The number needed to treat (NNT), i.e., the number of patients who must be treated to obtain one more therapeutic effect compared to the control group, was also calculated (26). NNT is the reciprocal of RD in mathematics, and the 95% confidence intervals for NNT are the reciprocal of the 95% confidence intervals for RD. The NNT tells us the estimated number of patients that need to be treated with the intervention to prevent an unfavorable event compared with the control group. For calculating RD and NNT from meta-analyses, we refer to the methods cited by Palazon-Bru (27).

The alpha level was set at 0.05, for a two-tailed test.

R statistical software version 4.0.3 (R Project for Statistical Computing, Vienna, Austria) was used to do all the calculations for the meta-analysis.

Interstudy heterogeneity was evaluated using the Q statistic of DerSimonian and Laird (24), and the relevance of heterogeneity was measured using the *I* ^2^ (28, 29).

We decided to follow the recommendations of the international Grade Guidance 24 on integrating of randomized and non-randomized studies (12).

### Trial sequential analysis

TSA was performed to assess whether the results regarding the primary outcome (primary or secondary prevention of diverticulitis) would be subject to type I or type II errors. TSA combines traditional meta-analysis methodology with repeating significance testing methods applied to accruing data in clinical studies (30). TSA basically calculates the relative risk reduction (RRR). TSA constructs monitoring boundaries to establish when an estimated effect is so convincingly large that the conclusions are unlikely to change with additional evidence. A model of variance-based diversity-adjusted information size was used for the TSA based on α = 0.05 and β = 0.20 (power of 80%) to be more conservative. The cumulative Z-curve of each cumulative meta-analysis was computed and plotted against the above monitoring boundaries. The crossing of the cumulative Z-curve into the trial sequential monitoring boundary for benefit suggests that a sufficient level of evidence has been reached, and no additional studies may be needed to demonstrate the superiority of the intervention. If the cumulative Z-curve does not cross any of the trial sequential monitoring boundaries, there is probably not sufficient evidence to reach a conclusion, and further studies may be needed.

Meta-analyses cannot be merely data pooling exercises, and TSA appears to be a useful tool for drawing non-biased conclusions (31, 32).

TSA was conducted using Trial Sequential Analysis software version 0.9.5.10 beta (Copenhagen Trial Unit, Centre for Clinical Intervention, Copenhagen, Denmark). All *p* values < 0.05 were considered statistically significant.

## Results

### Search findings

The initial combined search identified 1033 reports, and we excluded 305 because of the title or abstract. Of the remaining 726 articles, we excluded 280 for being not pertinent, 46 registered as reviews, 179 for being dedicated to surgery, 68 dealing with acute diverticulitis, and 66 referring to observational studies not fitting with the predefined PICO criteria. In this group, one cross-over study was excluded from the final analysis (33) due to a short follow-up (14 days). Another one was not considered due to a lack of sufficient information on the rate of diverticulitis relapse (34). A third compatible study was excluded due to a regimen of both rifaximin and mesalamine (35). Twenty-four papers were case reports, and 55 concerned the therapy of lower GI hemorrhage.

**Figure 1:**
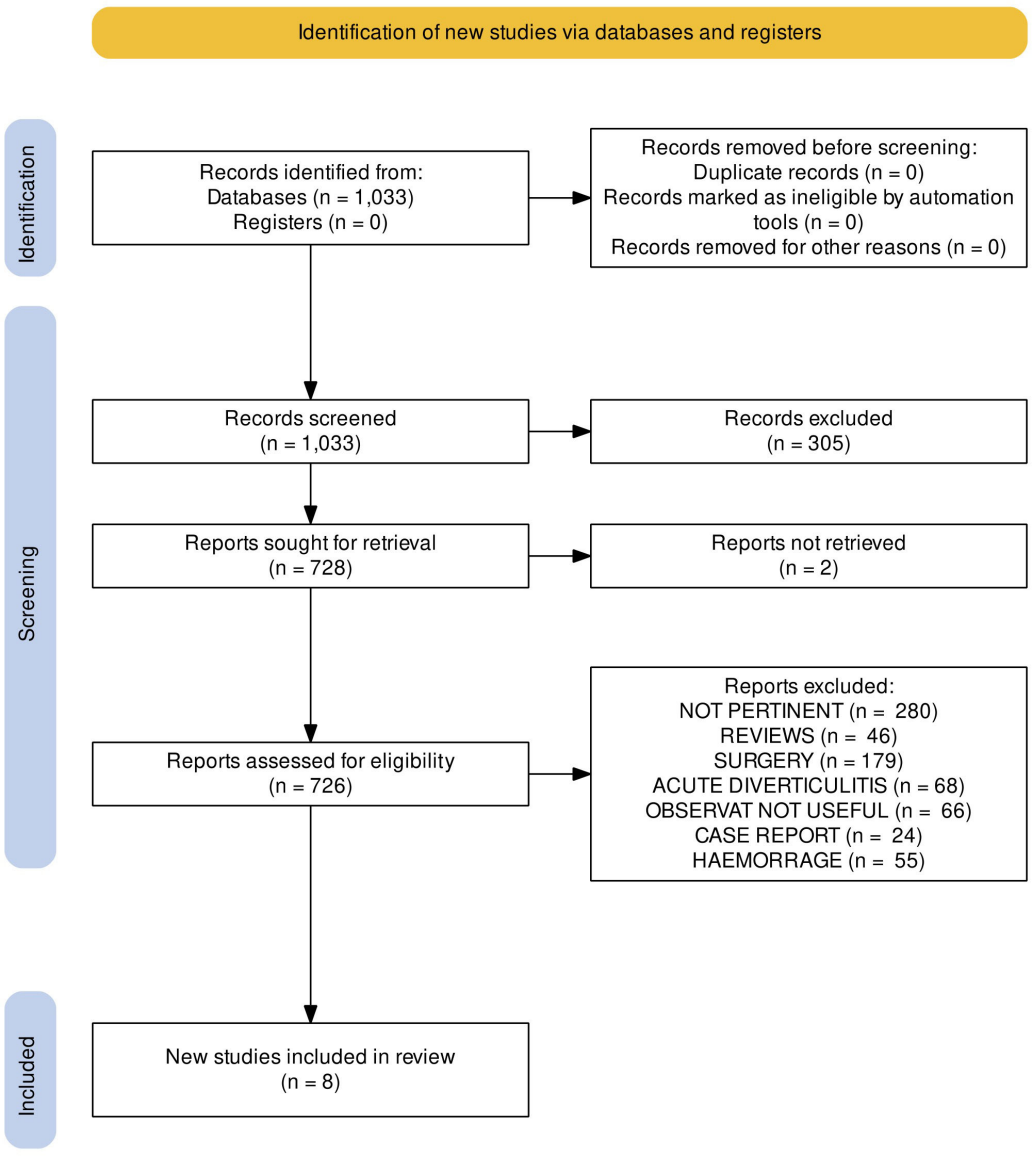
PRISMA flow diagram, according to PRISMA 2020 software.

This review is based on the results of eight studies (36–43) (see **Figure 1**).

A total of 3013 patients had been enrolled: 1568 were assigned to treatment with rifaximin, and 1445 to no treatment or mesalamine. There were five studies for preventing the first episode of diverticulitis (primary prevention, PP) (36–39, 43) and three for preventing diverticulitis recurrence (secondary prevention, SP) (40–42).

All studies for primary prevention were randomized trials (36–39), except one retrospective (43).

Two studies in the secondary prevention group (41, 42) were retrospective, and one was a prospective trial (40).

The characteristics of the studies are shown in **Table 1**. In all studies, the antibiotic used was rifaximin 400 mg b.d. for 7–10 days every month; all patients in both the treated group and control group received a standard dietary fiber supplement, unless in the Polish study, where we were not able to extract the information. In only one study, the control group received a placebo (37). In one study, control therapy included mesalamine 2,4 g/day for 10 days per month (41).

**Table 1:** Studies addressing non-absorbable antibiotic rifaximin in the prevention of diverticulitis.

Author	No. patients	Study design	Treatment	Study period (years)
PRIMARY PREVENTION				
Papi et al, 1992 (36)	217	RCT	glucomannan 2g + rifaximin*	1
			glucomannan 2g	
Papi et al, 1995 (37)	168	RCT	glucomannan 2g + rifaximin *	1	
			glucomannan 2g + placebo		
Latella et al, 2003 (38)	968	RCT	glucomannan 4g + rifaximin *	1	
			glucomannan 4g		
Di Mario et al, 2019 (43)	816	Observational	rifaximin* Symptomatic Tx@	8	
SECONDARY PREVENTION					
Lanas et al, 2012 (40)	165	RCT	rifaximin* plus fibers § fibers §	1	
Festa et al, 2016 (41)	124	Observational	dietary fibers Supp† + rifaximin* mesalamine ^	1	
Banasiewicz et al, 2017 (42)	248	Observational	rifaximin* control	1	

• Rifaximin 400 mg b.d. for 7-10 days each month for 12 months.

• † Dietary fiber Supplementation (20 g/die).

• @ Symptomatic Therapy: short-term course of fiber, spasmolytics, mesalamine, probiotics

• § 3.5 g of high-fiber Supplementation b.d.

• ^ Mesalamine 2.4 g/daily ten days a month

• Study design: RCT; randomized control trial.

All studies followed patients for up to one year, except for the Di Mario study (8 years) (43).

#### Quality appraisal

Regarding RCTs, there were some concerns due to possible biases for deviations from the assigned group and to missing data in all 5 RCTs. The randomization process was unclear in two of them (**Supplementary Data; Figure 1A**).

All 3 observational studies were in an area of low-risk bias (8 stars) (**Supplementary Data; Figure 1B**).

### Prevention of the first episode of diverticulitis (primary prevention)

**Figure 2:**
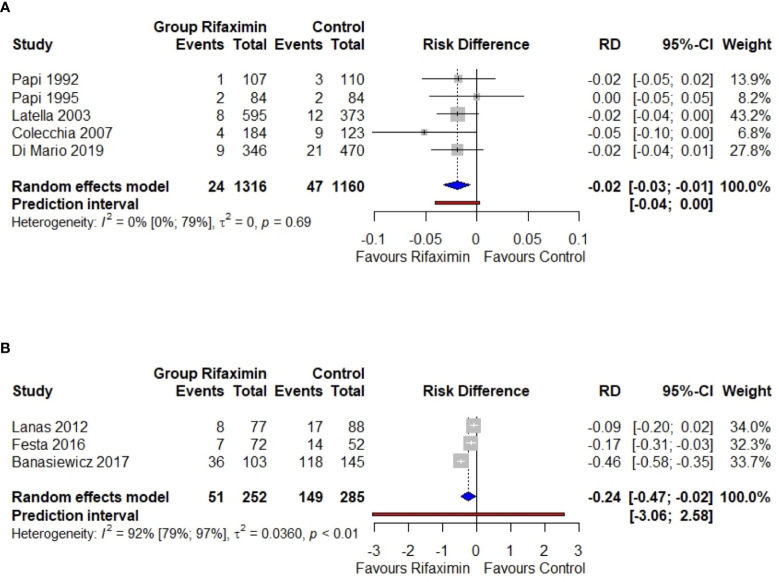
Meta-analysis regarding the risk difference between the rifaximin group and controls as the primary **(A)** or secondary **(B)** prevention of diverticulitis. RD, risk difference; 95%-CI, confidence intervals at 95%. The vertical line indicates the ‘no difference’ point between the two options. Squares represent the adjusted risk difference. Diamonds represent the pooled risk difference for all studies. Horizontal lines represent 95% CI.

We found 5 studies: 4 RCTs (36–39) and 1 observational study (43). The pooled risk of primary diverticulitis among SUDD untreated patients showed a moderate increase through the years: 3.0% (CI 95% 1.6–4.4%), 3.2% (CI 95% 1.4–5.0%), and 4.5% (CI 95% 2.6–6.3%) at 1, 2, and 8 years, respectively. The pooled risk difference was significative in favor of rifaximin (-1.9%, CI -0.6% to -3.3%). There was no evidence of heterogeneity (*I* ^2^ 0%) (**Figure 2A**).

**Figure 3:**
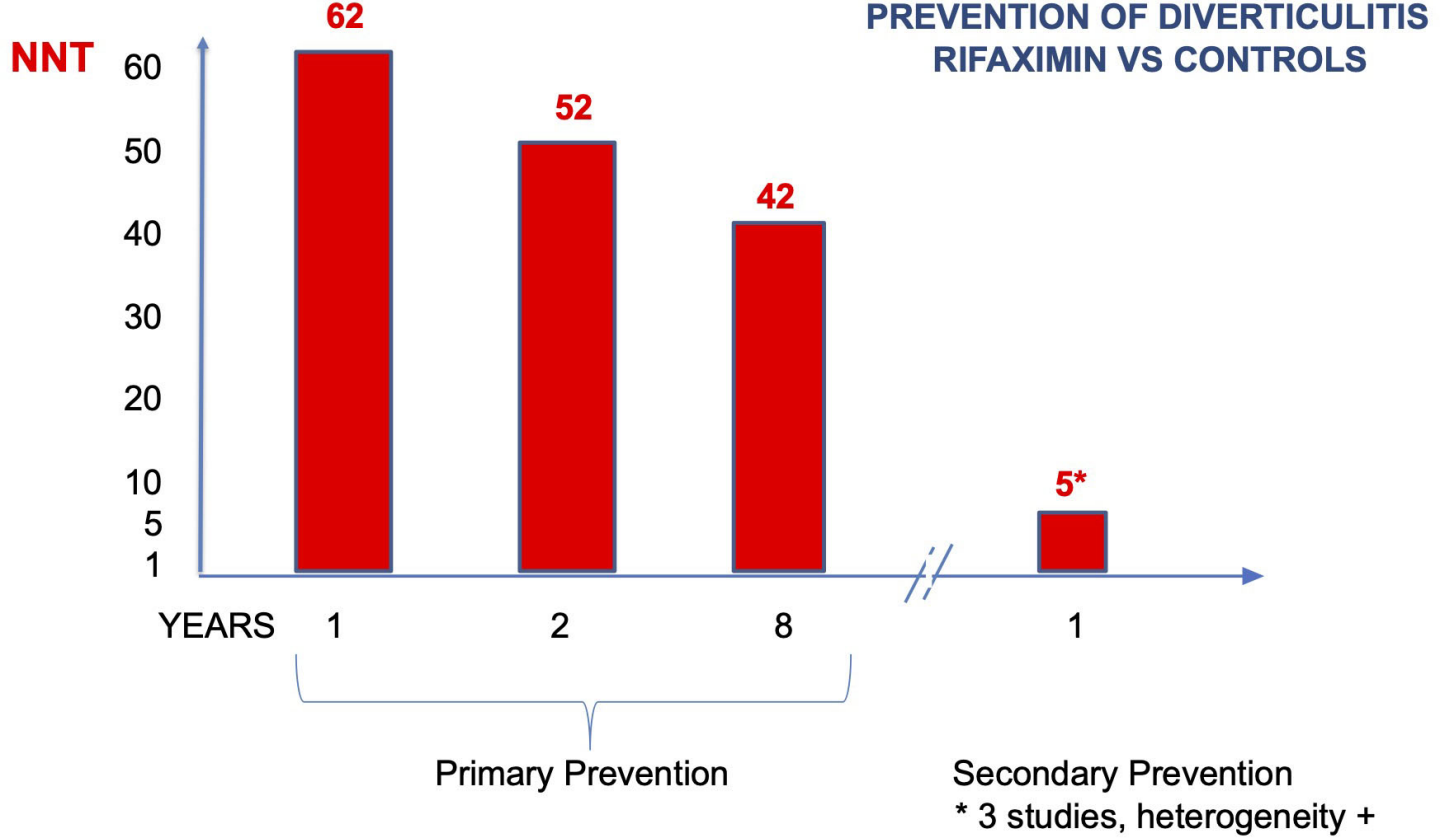
Number of patients who need to be treated to prevent diverticulitis (NNT) (primary and secondary prevention).

NNT resulted 62 (CI 95% 42–500), 52 (CI 95% 39–133), and 42 (CI 95% 31–96) at 1, 2, and 8 years, a slight reduction in time (**Figure 3**) (27). In summary, the use of rifaximin in SUDD patients really reduces the risk of diverticulitis, but the gain is clinically poorly relevant due to the low absolute risk.

#### Trial sequential analysis

**Figure 4:**
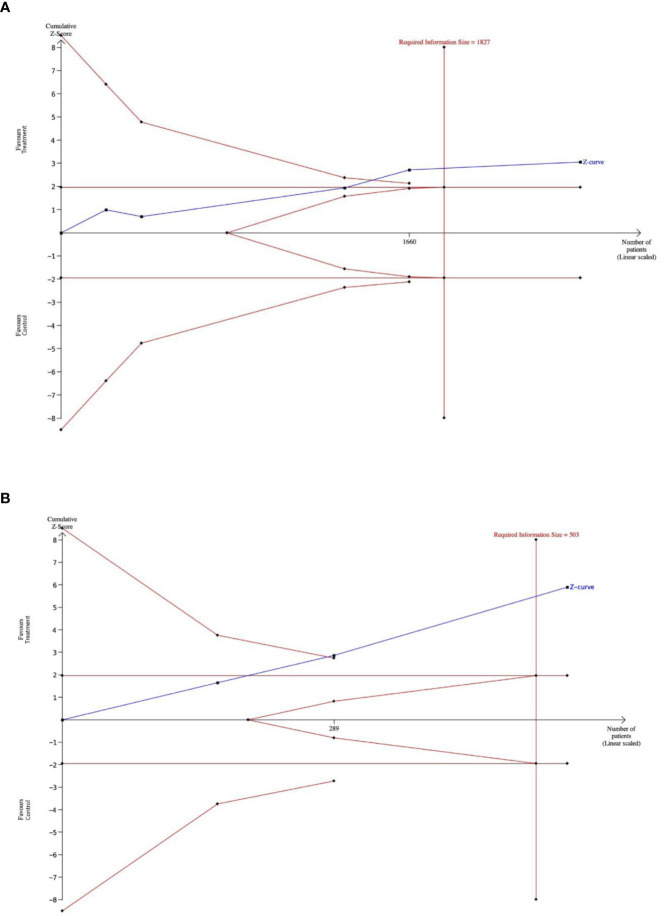
**(A)**. The outcome of a trial-sequential analysis comparing the rates of diverticulitis episodes in patients receiving primary prophylaxis with rifaximin versus patients who did not receive rifaximin. The diversity-adjusted required sample size (1,827 participants) was based on an alpha error of 5% and a beta error of 20. Cumulative z-curves were computed by a fixed effects model. (Alpha-boundaries are the external red lines) **(B)**. The outcome of a trial-sequential analysis comparing diverticulitis relapse rates in patients receiving secondary prophylaxis with rifaximin versus patients not receiving rifaximin. The diversity-adjusted required sample size (503 participants) was based on an alpha error of 5%, a beta error of 20%. Cumulative z-curves were computed by a random effects model. (Alpha-boundaries are the external red lines).

The type I error risk in our trial sequential analysis was set at = 0.05, with a power of 0.80 and a 25% expected relative risk reduction (RRR) linked to intervention. Under these premises, the required information size for the meta-analyzed estimate was 1,827. Thus, TSA confirmed the results obtained in the conventional meta-analysis. The Z-score curve (blue line) crossed both the required information size (vertical red line) and the conventional statistical significance boundary corresponding to a two-sided *p-value* of 0.05 (horizontal red lines), indicating that the observed reduction in the rate of primary diverticulitis in subjects taking rifaximin could be considered conclusive with the existing evidence (**Figure 4A**).

#### Conclusions

Current certainty of the evidence, when randomized control trials (RCTs) and non-randomized studies (NRS) (12) are included in evidence synthesis, suggests that rifaximin reduces the risk of primary diverticulitis in patients with SUDD. The grade level was moderate (**Supplementary Data; Figure 2**).

The resulting prediction interval, ranging from −0.04 to 0.00, can be interpreted as the 95% range of true RD expected in similar studies. The prediction interval contains values below zero, corresponding to a decrease in diverticulitis of at best ∼0.04 RD after rifaximin use compared with placebo.

### Prevention of further episodes of diverticulitis (secondary prevention)

We found 3 studies: 1 RCT (40) and 2 observational (41, 42).

Lanas (40) admitted 165 patients with a previous episode of diverticulitis in a multi-centric clinical trial. The authors randomized them to a group treated cyclically with Rifaximin 400 mg/bid (7 days monthly) and high-fiber supplementation and to a control group with high-fiber supplementation only. The authors followed their patients for one year, registering further episodes of diverticulitis. The recurrence rate of diverticulitis was 19.3% in the control group and 10.4% in the treated patients. Groups were comparable for age, sex, time since their first episode, and disease location.

Festa (41) referred to a retrospective cohort of patients who were followed in a dedicated out-patient clinic (*Mal.dive.* Clinic, from the Italian acronym *Malattia Diverticolare*) for 2 years. There were only two clinicians who were dedicated. They applied a pre-constructed form and prescribed 10 days monthly of rifaximin 400 mg/bid and high-fiber supplements, or mesalamine 2.4 g/bid and the same high-fiber supplement, to 124 patients, with a previous episode of diverticulitis. The two groups were comparable, also for ASA and NSAID use. Patients admitted to the study had a mean time of follow-up of 15 months. At one year, the recurrence rate was 26.9% in the control (mesalamine) group and 9.7% in the rifaximin group.

Banasiewic and his co-authors (42) observed retrospectively a large group of patients in the authors’ outpatient clinics. Patients were treated with 7 days of rifaximin 400 mg/bid, monthly, or with other medical therapy (controls). The two groups were comparable for age, sex, and disease duration. The authors registered a high risk of recurrent diverticulitis at one year of follow-up: 60% in the control group and 28% in the study group.

At one year, the risk difference was statistically significant in favor of rifaximin (-0.24, or -24%, CI -47 to -2%). Anyway, there was clear evidence of heterogeneity (*I* ^2^ 92%) (**Figure 2B**). If calculated, NNT resulted in 5 (CI 95% 4–7) (**Figure 3**).

In conclusion, the use of rifaximin in SUDD patients with a previous episode of diverticulitis may reduce the risk of a second episode (RD-24%). This finding is hampered by heterogeneity, due to a one-year higher risk of recurrence in the Polish patients.

#### Trial sequential analysis

In our trial sequential analysis, the type I error rate was set at 0.05, with a power of 0.80 and an expected RRR linked to an intervention of 35%. Under these premises, the required sample size for the meta-analyzed estimate was 503 patients. The Z-score curve (blue line) crossed both the required information size (vertical red line) and the conventional statistical significance boundary corresponding to a two-sided *p* value of 0.05 (horizontal red lines), indicating that the observed reduction in rate of secondary diverticulitis in subjects taking rifaximin could be expected in further studies (**Figure 4B**).

#### Conclusions

Certainty of the evidence, when randomized control trials (RCTs) and non-randomized studies (NRS) (12) are included in evidence synthesis, suggests that rifaximin might reduce the risk of diverticulitis relapse in patients with SUDD. Anyway, the GRADE level was considered low (**Supplementary Data Figure 3**).

The resulting prediction interval, ranging from −3.06 to 2.58, can be interpreted as the 95% range of true RD to be expected in future similar studies. The prediction interval contains values below zero, which correspond to a decrease in diverticulitis of at best ∼3.06 RD after rifaximin use compared with placebo. But it also contains values above zero, which means that the rifaximin may exhibit no or even a harmful effect (RD>0) in some settings, with a 95% worst case increase in RD of 2.58.

## Discussion

Diverticulosis of the colon develops in most individuals in western countries with increasing age and tends to remain asymptomatic (44, 45). Diverticulosis *per se* cannot be considered a disease. The term “diverticular disease” implies that there are symptoms related to the diverticula.

Most diverticulosis cases remain asymptomatic: only about 4% of patients with an endoscopic diagnosis of diverticulosis develop diverticulitis (46).

However, admissions for diagnosis code diverticulitis are on the increase: in the USA alone, the increase in the admissions rate in 2015 was +21% compared to 2003, with a total aggregate cost of between $2.2 and $2.6 billion and a hospital mortality rate of 0.5% (46).

Two very large European multicenter studies (47, 48) suggest that relapse after diverticulitis is relevant.

Binda’s Italian study reports on a follow-up of 320 patients treated with antibiotics in 17 hospitals after discharge for diverticulitis. In a comparable follow-up period (10.7 years), 25% of patients had relapse of symptoms requiring re-admission. The risk of surgery jumped to 17% (47).

Broderick-Villa reports on the history of 2,366 patients hospitalized for diverticulitis in the Kaiser system. At a median follow-up of 8.9 years, diverticulitis had recurred in 13.3% of patients (48).

A real-world Italian study recently published confirmed an increase in hospital admissions from 2011 to 2014 (+12%) (49). In one year, 8.2% of patients treated non-operatively were readmitted for diverticulitis. Most important, acute episodes of diverticulitis involved a 1.2% risk of mortality in patients over the age of 65.

A recent paper from a national study in Sweden on 97.850 cases shows that diverticulitis strongly elevates mortality vs controls by some 27% (50).

So, any measure to reduce the impact of diverticulitis on hospitalization and mortality should be welcomed.

Relevant evidence indicates that dietary fiber, especially the insoluble fiber found mostly in fruits and vegetables, decreases the risk of diverticula development (51). The protective effect of dietary fiber would make stools bulkier, which would increase the size of the colon, lower intraluminal pressures, and shorten the time it takes for the colon to move (52).

Both experimental and clinical data show that the non-absorbable antibiotic rifaximin has a broad-spectrum antibacterial action, covering gram-positive and gram-negative aerobic and anaerobic bacteria (53, 54).

Dietary fiber and non-absorbable antibiotics, such as rifaximin, interact for the treatment of diverticular disease, as rifaximin has been reported to improve the clinical benefits on symptoms of dietary fiber in SUDD patients. Treatment with rifaximin plus fiber supplementation is effective in obtaining symptom relief at 1 year. The pooled RD for *complete* symptom relief in favor of the rifaximin group was 29.0% (95% CI 24.5% to 33.6%; P < 0.0001; NNT = 3) (13).

The aim of our meta-analysis was dedicated to evaluating the long-term efficacy of administration of rifaximin in preventing diverticulitis in patients with SUDD. Including both RCTs and non-randomized studies in a systematic review has generated controversy and diverse opinions (55). We followed the GRADE recommendations for evidence syntheses to get the most useful information from the different types of studies used in health syntheses (12).

Rifaximin significantly reduced the risk of the first episode In SUDD patients (-1.9%, CI -6 to -3%). There was no evidence of heterogeneity (*I* ^2^ 0%). GRADE certainty of evidence was moderate. The result is confirmed by TSA, which shows that further investigations in the field are probably useless. Thus, rifaximin reduces the risk of the first episode of diverticulitis in patients with SUDD. Anyway, this finding has a very limited clinical relevance due to the high number of patients to gain a single episode of diverticultis (NNT of 62, 52, and 42 at 1, 2, and 8 years).

Rifaximin could reduce the risk of a second episode in SUDD patients (-24%, CI -47 to -2%). At 1 years, the NNT was 5. However, there was evidence of heterogeneity in pooling (*I2* 92%), and definite conclusions are blocked. This finding is due to a higher risk of recurrence in the Polish patients (81%). The resulting prediction interval suggests that the effect in a new study may be even the exact opposite of the summary point estimate of the meta-analysis, that is, an increase of 0.24 instead of a decrease of 0.51. So, the GRADE certainty of the evidence resulted in a low, and TSA suggests that more patients should be admitted for further studies (up to 503).

## Limitations

### Primary prevention

This meta-analysis has some limitations. The study is limited by the quality of the included studies. This could lead to an overestimation of the treatment effect of rifaximin. Three studies were RCTs (36–38), and one was an observational investigation (43). Blinding and a placebo-controlled group were guaranteed in one study only (37). Furthermore, the definition of diverticulitis was not pre-defined in all studies. Consequently, we asked the authors to reconsider including diverticulitis only in cases of hospitalization, and to recalculate the cases accordingly. This occurred in three studies (36, 37, and 39).

Anyway, heterogeneity was not observed. Following the prediction interval, the benefit of rifaximin to prevent diverticulitis can be guaranteed in further studies.

### Secondary prevention

The second meta-analysis includes only 3 studies (40–42), and only one was a randomized trial (40). Heterogeneity was observed, limiting the value of the statistical result. Following the prediction interval, the benefit of rifaximin to prevent recurrent diverticulitis cannot be guaranteed in further studies.

## Conclusions

A rifaximin regimen of seven to ten days per month resulted in a consistently better outcome in terms of the appearance of diverticulitis. The evidence is definitive regarding primary prevention: while reducing symptoms (13), rifaximin could reduce the risk of a first episode of diverticulitis. However, the cost-benefit ratio is questionable, and rifaximin cannot be recommended for all patients with SUDD. The quality of the evidence is moderate (i.e.: further research is likely to have an important impact on confidence in the estimate of effect and may change the estimate).

Rifaximin might reduce the risk of a second episode with a good ratio. But the quality of evidence is currently low, due to the low number of studies, and the associated risks of bias and heterogeneity (that is: further research is very likely to have an important impact on confidence in the estimate of effect and is likely to change the estimate).

Rifaximin should better be studied in a prospective trial for primary and secondary prevention. The required sample size for further RCTs can be estimated as the difference between the required information size and the number of people already recruited into the previous trials (56).

Furthermore, we need to concentrate the analyses on patients with SUDD who have a higher prior probability of developing diverticulitis. This could be accomplished by identifying patients who have risk factors that may aggravate their clinical history (e.g., obesity, sedentary lifestyle, use of NSAIDs or aspirin, immunosuppressive therapy). Possibly, a subgroup of patients could be identified for whom the cost-benefit ratio f or rifaximin use in prevention could be more favorable (57).

An economic analysis on the long-term prescription of rifaximin must also be performed and is currently under development. In Italy, a monthly cycle of rifaximin costs 16.64 euros and is reimbursed by the National Health System. Preliminary findings have already been published (49).

## Data availability statement

The raw data supporting the conclusions of this article will be made available by the authors, without undue reservation.

## Author contributions

MK, AEM, substantial contributions to the conception or design of the work, or the acquisition, analysis, or interpretation of data for the work, GN,SC drafting the work or revising it critically for important intellectual content. All authors contributed to the article and approved the submitted version.
