# Supplementary material for: Preventing acute diverticulitis. any roles for non-absorbable antibiotics? in search of evidence: a systematic review, meta-analysis, and trial sequential analysis
Source: Front Gastroenterol (Lausanne). 2023 May 1;2:1170271. doi: 10.3389/fgstr.2023.1170271 (PMC12952390; doi:10.3389/fgstr.2023.1170271)

Figure 1. The revised tool to assess the risk of bias in the five randomized trials (RoB 2). and the Newcastle-Ottawa Scale (NOS) for assessing the quality of the three non-randomized studies.

**PANEL A: ROB2**


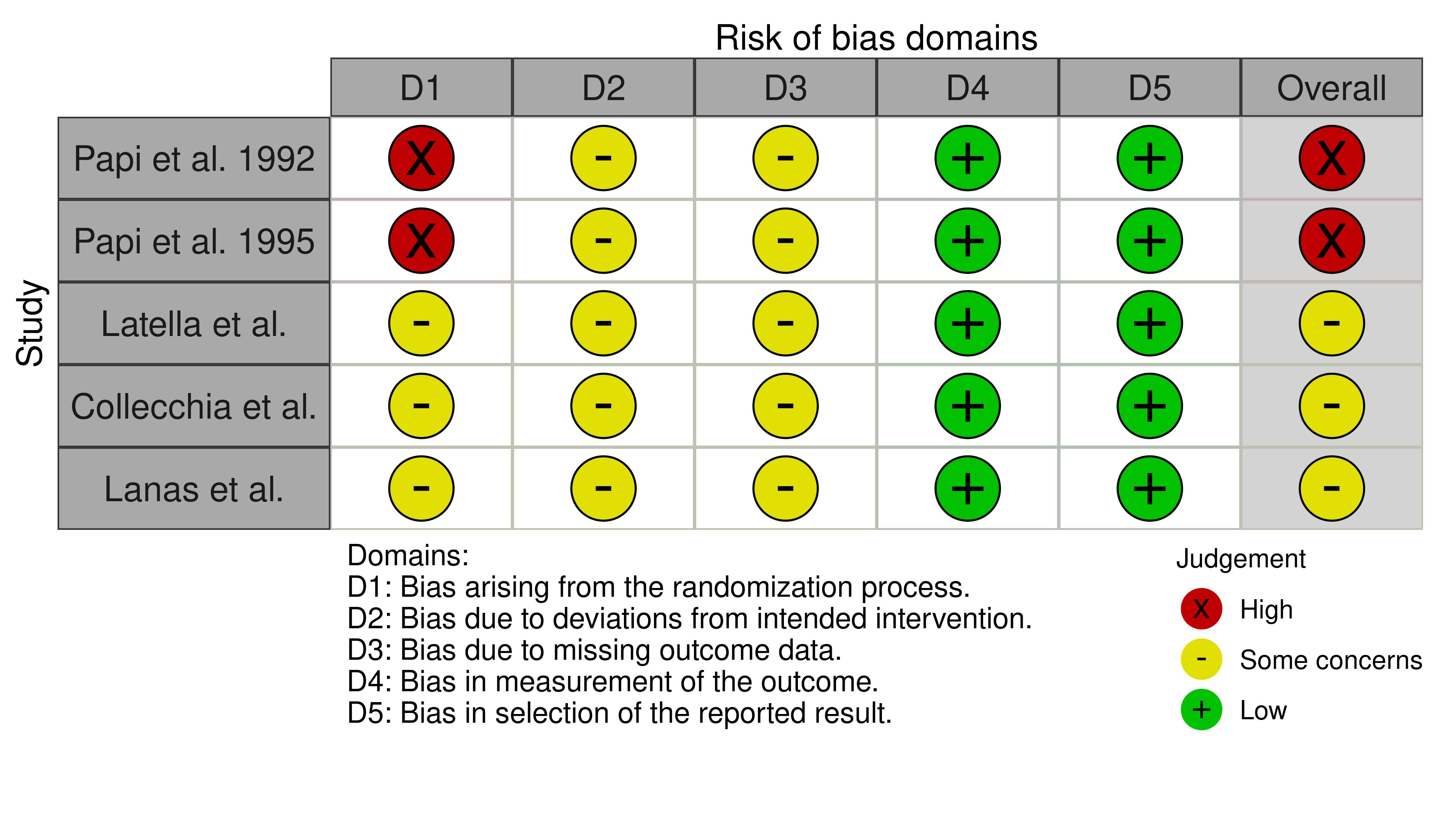


**PANEL B: NEWCASTLE OTTAWA SCALE (NOS)**

| Cohort studies | | | | | | | | | |
| --- | --- | --- | --- | --- | --- | --- | --- | --- | --- |
| Study | SELECTION  MAX 4 | | | | COMPARABILITY  MAX 2 | OUTCOME  MAX 3 | | | TOTAL STAR RATING  UP TO 9 |
|  | Representativeness  of the  exposed cohort | Selection  of the  non-exposed cohort | Ascertainment of exposure | Outcome  not present  at start of  study | Comparability of cohorts at baseline | Assessment of outcome | Follow-up long enough | Follow-up complete | Assessment of  bias risk |
| Festa et al. |  |  |  |  |  |  |  |  | 8 Low risk of bias |
| Banasiewicz et al. |  |  |  |  |  |  |  |  | 8 Low risk of bias |
| Di Mario et al. |  |  |  |  |  |  |  |  | 8 Low risk of bias |

FIGURE 2**.**

Prevention of the first episode of diverticulitis after assessment of the GRADE certainty of evidence when non-randomized studies (NRS) and randomized control trials (RCTs) are included in an evidence synthesis (modified from 44)

###
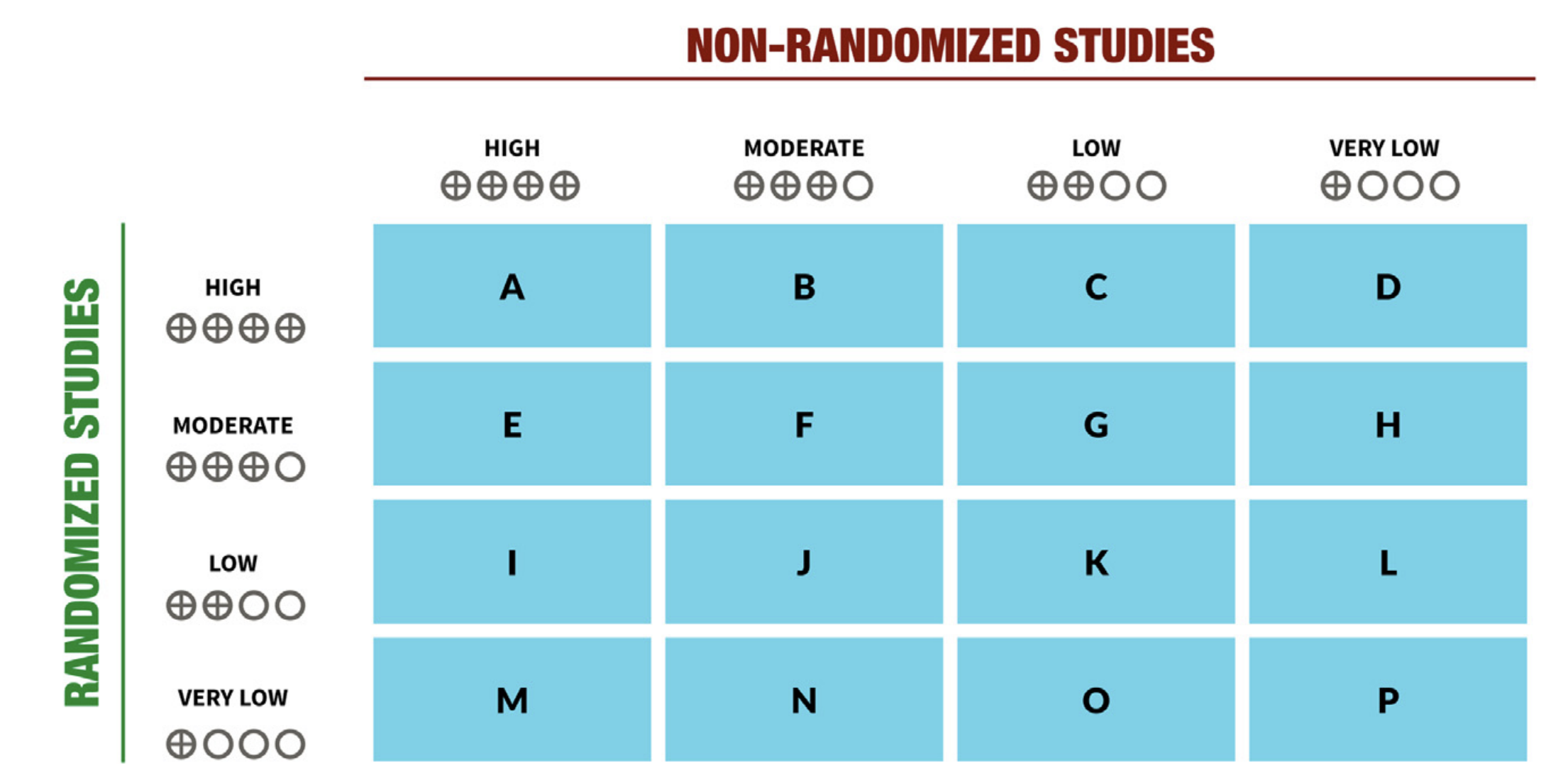


### FIGURE 3.

Prevention of further episodes of diverticulitis after assessment of the GRADE certainty of evidence when NRSI and RCTs are included in an evidence synthesis (modified from 44).

###
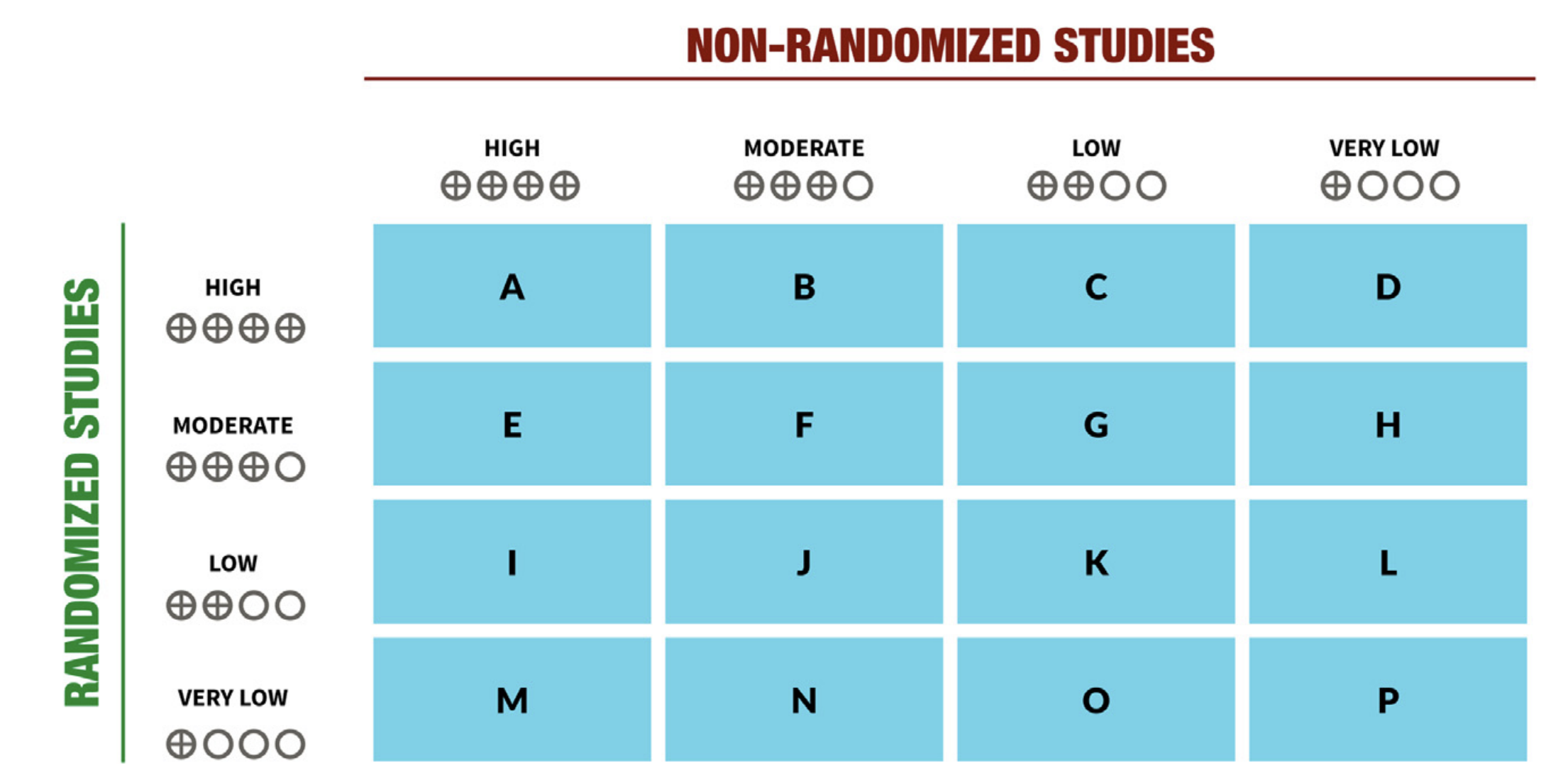

Supplement: Supplementary file 1 [file DataSheet_1.docx]
